# Skewed MHC Class I Peptide Presentation in Psoriatic Arthritis

**DOI:** 10.3390/ijms27167317

**Published:** 2026-08-16

**Authors:** Sara Comdühr, Thorben Sauer, Silja Deckert, Silke Pitann, Marco Schäfer, Timo Gemoll, Sabrina Arnold, Sebastian Klapa, Gabriela Riemekasten, Diamant Thaçi, Hanna Graßhoff, Peter Lamprecht

**Affiliations:** 1 Department of Rheumatology and Clinical Immunology, University of Luebeck, Ratzeburger Allee 160, 23538 Lübeck, Germany; 2 Section for Translational Surgical Oncology and Biobanking, Department of Surgery, University of Luebeck, Ratzeburger Allee 160, 23538 Lübeck, Germany; 3 HLA-Laboratory, Stefan Morsch Foundation, Dambacher Weg 3–5, 55765 Birkenfeld, Germany; 4 Institute of Experimental Medicine, Christian-Albrechts-University of Kiel, Kopperpahler Allee 120, 24119 Kronshagen, Germany; 5 Institute and Comprehensive Center for Inflammation Medicine, University of Luebeck, Ratzeburger Allee 160, 23538 Lübeck, Germany

**Keywords:** psoriatic arthritis, hnRNP A1, immunopeptidome, MHC class I, glutamic acid

## Abstract

Given the strong association between major histocompatibility complex (MHC) class I and psoriatic arthritis (PsA), and the role of the MHC in antigen presentation potentially driving immunopathology, we performed mass spectrometry-based immunopeptidome analysis to identify MHC class I-bound peptides in PsA. MHC class I expression on circulating immune cells was determined by flow cytometric analysis. MHC class I-bound peptides were isolated via immunoaffinity purification and identified by mass spectrometry in PsA patients and healthy controls. MHC class I expression was increased on circulating immune cells in PsA. MHC class I immunopeptidome analysis revealed a high prevalence of peptides of nine-amino-acid length originating from intracellular source proteins, typical for MHC class I antigen presentation. Analysis of the MHC class I immunopeptidome showed a greater relative abundance of glutamic acid at position 2 in nonamers from PsA patients, but not controls. This finding was further corroborated by an unsupervised clustering analysis showing five different clusters in healthy individuals and six in PsA, with one featuring higher abundance of glutamic acid at position 2. The glutamic acid-containing PEQLRKLFI peptide from the heterogeneous nuclear ribonucleoprotein A1 (hnRNP A1) was exclusively identified in PsA patients both via MHC class I immunopeptidome analysis and via PEPperMap epitope mapping. In PsA, MHC class I expression is increased on circulating immune cells, and the MHC class I immunopeptidome is perturbed, with disease-related skewing towards greater relative abundance of glutamic acid at position 2 in nonamers and presentation of a unique peptide motif from the putative autoantigen hnRNP A1.

## 1. Introduction

Psoriatic arthritis (PsA) is a chronic immune-mediated disease characterized by T cell-driven epidermal hyperplasia and synovio-enthesal inflammation, eventually causing bone erosion and disabling destructive joint disease in up to 50% of patients. As part of the psoriatic disease spectrum, PsA affects approximately 30% of patients with psoriasis (PsO) [1]. Psoriatic disease is associated with different major histocompatibility complex (MHC) class I alleles and gene variants of the antigen-processing endoplasmic reticulum aminopeptidases ERAP1 and ERAP2 [1,2]. MHC class I molecules mediate presentation of peptides originating from intracellular source proteins, which are translocated onto MHC and displayed on the cell surface for antigen recognition by CD8+ T cells [2]. The MHC class I-corresponding human leukocyte antigen (HLA) allele HLA-C*06 is a strong genetic risk factor for PsO, but not for PsA [1,2,3]. By contrast, the HLA alleles HLA-B*08, HLA-B*27, HLA-B*38, and HLA-B*39 are associated with an increased risk for different PsA subtypes, i.e., sacroiliitis and dactylitis with the former two and peripheral arthritis with the latter two alleles [3]. Accordingly, early epidermal infiltration of cytotoxic CD8+ T cells (Tc) is essential for the development of PsO rather than CD4+ T helper cell infiltration (Th) [4]. Moreover, resident interleukin (IL)-17 producing epidermal CD8+ memory T cells (Tc17) retain site-specific disease memory as the origin of recurrent psoriatic skin lesions [5]. Based on their cytokine production, increased frequencies of circulating Th1, Th17, and Th22 cells and enrichment of Th1, Th9, Th17, and Th22 cell populations and their CD8+ cytotoxic counterparts (Tc1, Tc17, Tc22) have been reported in psoriatic lesions [4,5]. By contrast, T-cell-mediated immunopathology is less well characterized in PsA. CD8+ T cells play a role in the transition from PsO to PsA, and resident Tc17 cells within synovial fluid and tissue may contribute to disease persistence [6].

Psoriatic disease displays features of both an autoinflammatory and autoimmune disease [7]. CD8+ T cells and/or antibodies with specificity for various melano- and keratinocyte-derived autoantigens have been reported in 20–80% of patients with psoriatic disease, but the immunopathological role of these autoantibodies and autoantigen-specific CD8+ T cells remains to be further elucidated [2,7,8]. Of note, analysis of naturally processed peptides that are translocated from the cell interior to the cellular surface and presented via MHC class I alleles from antigen-presenting cells (APC) for recognition by CD8+ T cells via their T cell receptor (TCR) provides a means for the identification of source proteins from which those peptides are derived and thus, antigens potentially inducing specific T cells responses [9]. The term immunopeptidome encompasses the entirety of MHC-bound peptides on the surface of antigen-presenting cells for recognition by CD8+ T cells, some of which eventually elicit and sustain T-cell responses [2,10,11]. Currently, mass spectrometry (MS)-based analysis of the immunopeptidome is the only unbiased method that allows direct identification of peptides naturally processed and presented via the MHC [10,11,12]. MS-based immunopeptidome analysis has been widely used for the identification of viral epitopes to improve vaccination [11] and tumor-associated antigens for the design of epitope-specific cancer immunotherapies [12]. Recent studies have focused on the use of MHC class II immunopeptidome analysis to identify key autoantigens in autoimmune diseases like rheumatoid arthritis and celiac disease [13,14,15,16,17,18]. A few studies also analyzed the MHC class I immunopeptidome in type 1 diabetes and multiple sclerosis [19,20,21]. Most of the studies were performed from cell lines or tissues. However, the technology has not been used to study MHC class I-bound peptides on circulating immune cells in PsA so far. Given its strong MHC class I association and the role of MHC class I molecules in antigen presentation potentially driving PsA immunopathology, we therefore determined MHC class I expression on circulating immune cells and performed MS-based immunopeptidome analysis to identify MHC class I-bound peptides and their intracellular source proteins, including putative autoantigens.

## 2. Results

### 2.1. Patients’ Characteristics and Prevalence of HLA Alleles

Patients’ characteristics are provided in Supplementary Tables S1 and S2. There was an overlap of four patients in whom both flow cytometry and LC-MS/MS-based immunopeptidome analysis were performed. Overall, slightly more women than men were included in this study. Patients were middle-aged and had a body mass index (BMI) of around 30. The average disease duration exceeded five years. At the time of analysis, patients had low cutaneous disease activity as determined by BSA and PASI. Regarding musculoskeletal manifestations, 90% of the patients had peripheral arthritis, and about a quarter had axial manifestations and dactylitis, respectively. Overall disease activity was moderate as determined by DAPSA, DAS28-CRP, and BASDAI. One patient had high disease activity. All patients were receiving treatment with either biologic or targeted synthetic disease-modifying anti-rheumatic drugs (bDMARDs, tsDMARDs) (Supplementary Tables S1 and S2).

The prevalence of individual HLA allele frequencies in PsA patients and healthy controls is shown in Supplementary Table S3. In this study, the PsA-associated alleles HLA-B*08:01 and HLA-B*27:02 [3] were detected in the group of PsA patients, but not in the group of healthy donors. The PsO-associated allele HLA-C*06:02 [1,2,3] was found with similar frequency in PsA patients and healthy individuals. Individual HLA-A, -B and -C alleles for each PsA patient and healthy control are listed in Supplementary Table S4.

### 2.2. Increased MHC Class I Expression of Circulating Immune Cells in PsA

**Figure 1:**
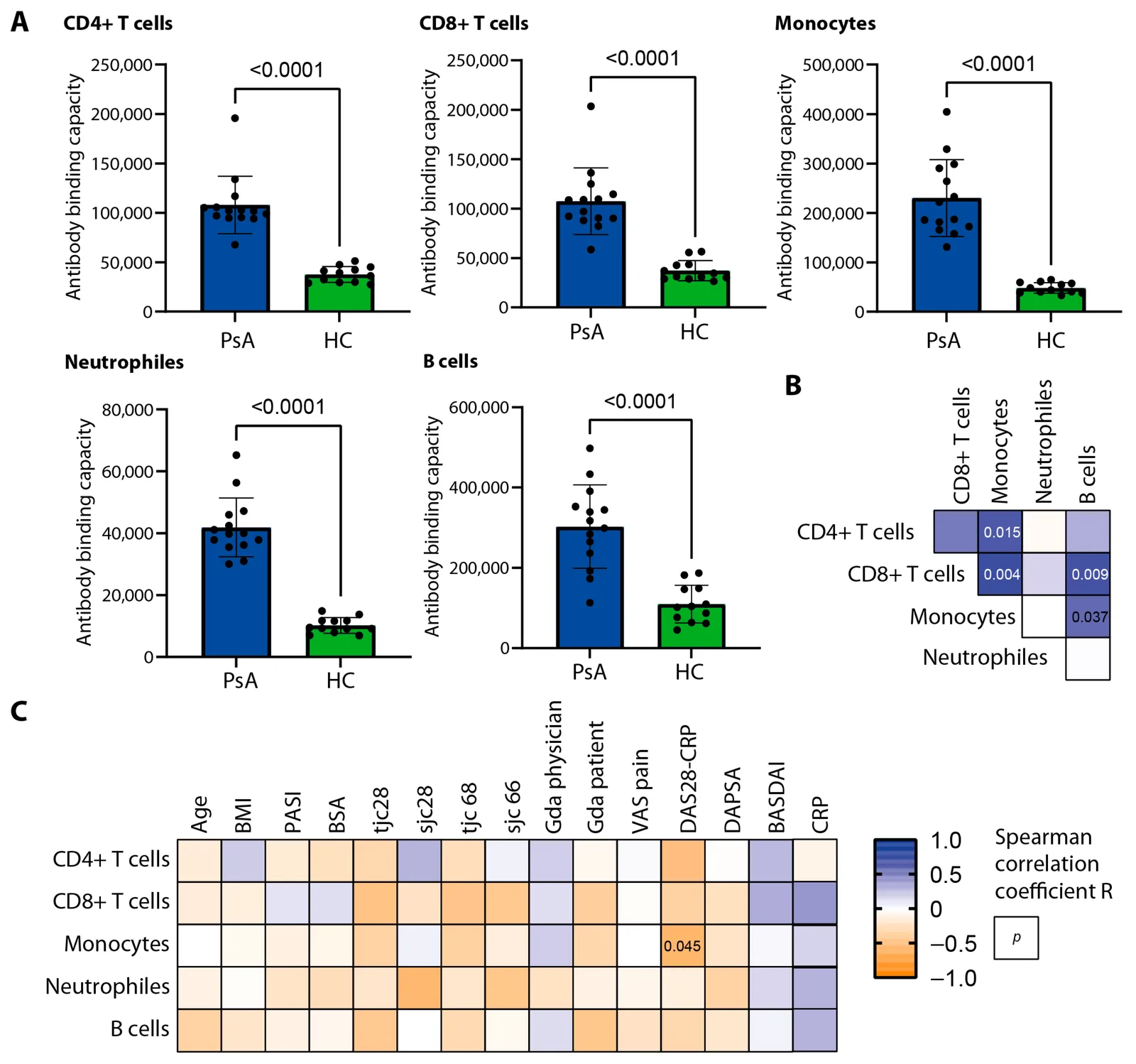
MHC class I cell surface expression in PsA patients (*n* = 14) and healthy controls (*n* = 12). (**A**) Flow cytometry data analysis of MHC I cell surface expression on CD4+ and CD8+ T-cells, B cells, monocytes and neutrophils. Error bars represent SD. Statistical comparison of antibody-binding capacity between immune cells in PsA patients and healthy controls was performed using the Mann–Whitney *U* test. Respective unadjusted *p*-values are shown. (**B**) Color-coded correlation heatmap displaying Spearman correlation coefficient R between MHC class I cell surface expression on CD4+ and CD8+ T-cells, B cells, monocytes and neutrophils. A positive Spearman correlation coefficient R is presented in blue, a negative in orange. Unadjusted *p*-values < 0.05 are inserted in the graph. (**C**) Color-coded correlation heatmap displaying Spearman correlation coefficient R between MHC class I cell surface expression on different cell types and demographic and clinical characteristics. A positive Spearman correlation coefficient R is presented in blue, a negative in orange. Unadjusted *p*-values < 0.05 are inserted in the graph. Abbreviations: BASDAI, Bath Ankylosing Spondylitis Disease Activity Index; BMI, body mass index; CRP, C-reactive protein, DAPSA, Disease Activity in Psoriatic Arthritis; DAS28-CRP, Disease Activity Score 28-C-reactive protein; HC—healthy control; MHC, major histocompatibility complex; PASI, Psoriasis Area and Severity Index; PsA, psoriatic arthritis; SD, standard deviation, SJC, swollen joint count (number denotes evaluation of 28 or 66 joints); TJC, tender joint count (number denotes evaluation of 28 or 68 joints); VAS, visual analog scale.

MHC class I expression on circulating immune cells was determined by flow-cytometric analysis using QSC beads to quantify MHC class I molecules on the cell surface. An increase in MHC class I expression was found on CD4+ and CD8+ T cells, B cells, monocytes, and neutrophils (Figure 1A). Next, we analyzed correlations between MHC class I cell-surface expressions on different circulating immune cell populations. A strong correlation was observed between CD4+ as well as CD8+ T cells and monocytes, between CD8+ T cells and B cells, and between B cells and monocytes (Figure 1B). Finally, we examined correlations between MHC I cell surface expression on immune cells and clinical parameters. A strong inverse correlation was found between MHC class I expression on monocytes and DAS28-CRP (Figure 1C).

### 2.3. Immunopeptidome Analysis Reveals Predominantly Nonamers from Intracellular Components as Source Proteins of MHC Class I-Bound Peptides

**Figure 2:**
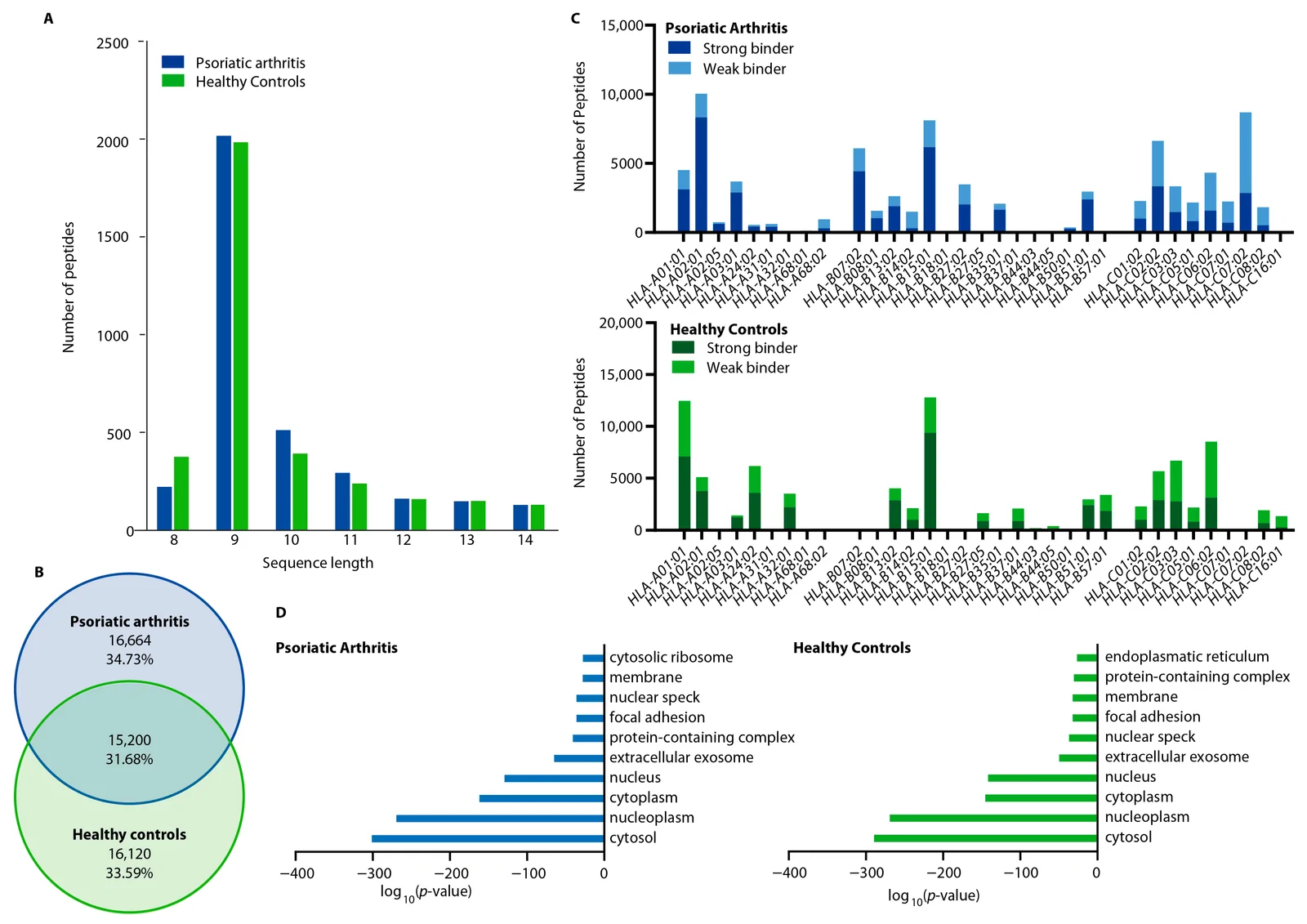
Length distribution, number of peptides, predicted binding affinity, and Gene Ontology (GO) analysis of cellular source proteins of MHC class I-bound peptides in PsA patients (*n* = 10) and healthy controls (*n* = 6). (**A**) Length distribution of MHC class I-bound peptides. Bar charts show the number of peptides of the respective peptide sequence length ranging from a length of 8 to 14 amino acids. (**B**) Venn diagram showing the number and percentage of peptides shared among PsA patients and healthy controls and those unique to PsA patients or healthy controls, respectively. (**C**) Number of peptides predicted as strong and weak binders among MHC class I-bound peptides isolated from individual HLA-A, -B and -C alleles are presented for PsA patients (blue) and healthy controls (green). HLA binding prediction was performed with NetMHCpan 4.0 [22]. By default, %Rank < 0.5% and %Rank < 2% thresholds are considered for detecting strong and weak binders, respectively. (**D**) GO categories of cellular source protein origin of isolated MHC class I peptides using DAVID Functional Annotation Bioinformatics Microarray Analysis. Bar graphs show top 10 enriched terms with respect to their log10 adjusted *p* value (Fisher’s exact test, Benjamini–Hochberg correction). Abbreviations: GO, gene ontology; MHC, major histocompatibility complex; PsA, psoriatic arthritis.

MHC class I-bound peptides were isolated from PBMC using immunoaffinity purification and identified by LC–MS/MS for immunopeptidome analysis. The overall peptide length distribution of isolated MHC class I-bound peptides showed the highest abundance for peptides 9 amino acids in length, i.e., nonamers (Figure 2A). Among the isolated MHC class I-bound peptides, some were found in both PsA patients and healthy controls, whereas others were present only in PsA patients or only in healthy individuals (Figure 2B). The number of peptides predicted to be strong or weak binders for individual *HLA* alleles varied, with both strong and weak binders found among MHC class I allele-bound peptides in individual PsA patients and healthy individuals (Figure 2C). Gene ontology (GO) enrichment analysis identified different intracellular components as source proteins of MHC class I-bound peptides in PsA patients and healthy individuals (Figure 2D).

### 2.4. Greater Relative Abundance of Glutamic Acid at Position 2 in MHC Class I-Bound Nonamers in PsA

**Figure 3:**
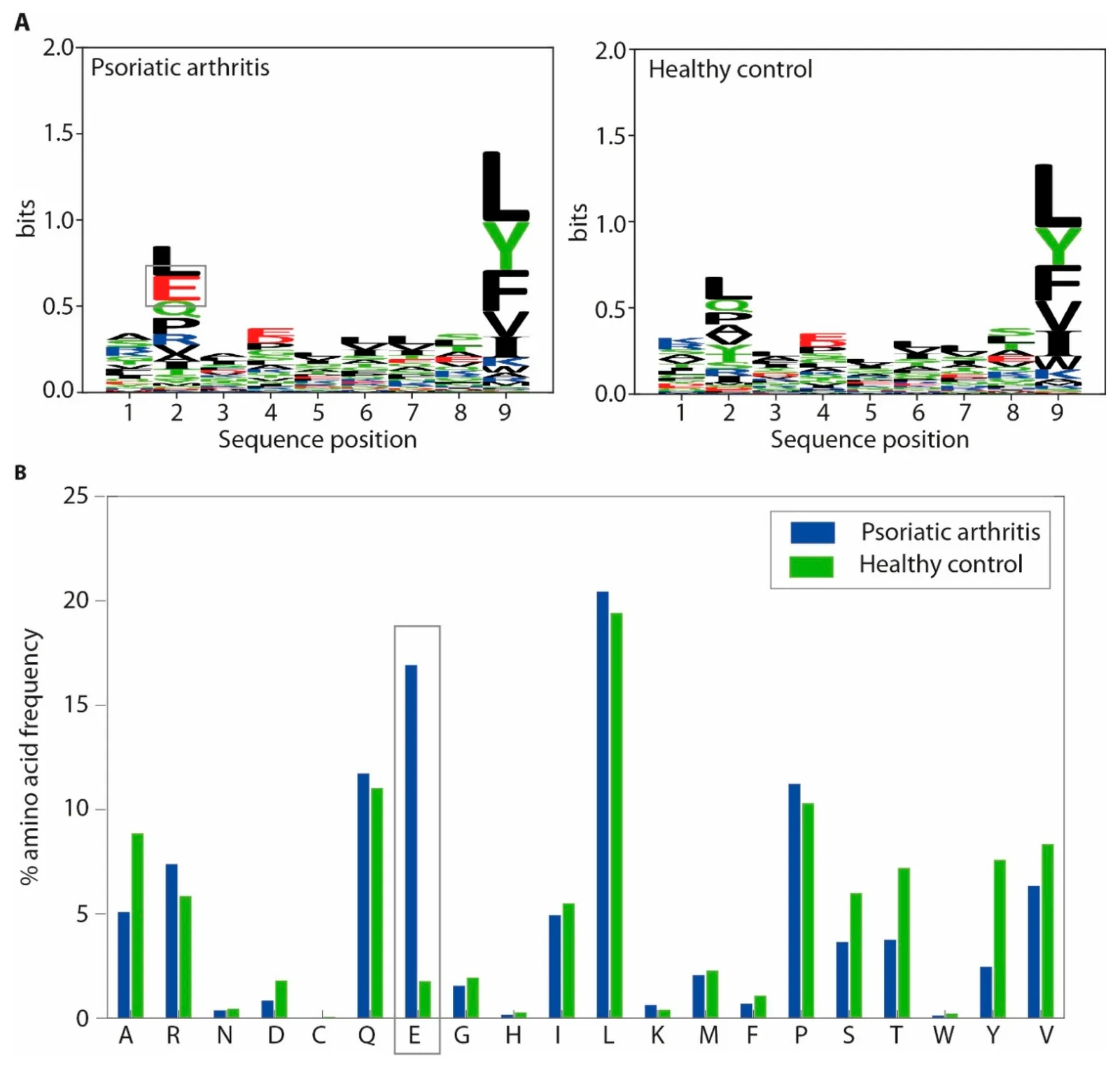
Sequence logos of MHC class I-bound nonamers isolated from PsA patients (*n* = 10) and healthy controls (*n* = 6) were generated using MHCpLogics. (**A**) Sequence logos of all identified nonamers (one letter amino acid code [23]) for PsA patients (**right**) and healthy controls (**left**). The size of the letter represents the relative abundance of each amino acid. The sequence logos apply a simplified chemistry-based color scheme in which acidic residues appear in red, basic residues in blue, hydrophobic residues in black, and aromatic as well as small neutral residues in green. At position 2, patients with PsA display a greater relative abundance of glutamic acid (black box). (**B**) Frequency of amino acids at position 2 of MHC class I-bound peptides. A difference regarding the frequency of glutamic acid at position 2 between PsA patients (blue) and healthy controls (green) is marked (box). Abbreviations: MHC, major histocompatibility complex; PsA, psoriatic arthritis.

Relative abundance of amino acids at positions 1 to 9 in MHC class I-bound nonamers identified by LC-MS/MS-based analysis was determined in PsA patients and healthy controls. A key difference between patients with PsA and healthy controls was the greater relative abundance of glutamic acid at position 2 in those nonamers in PsA (Figure 3A). To validate this finding, the frequency of each amino acid at position 2 in MHC class I-bound nonamers was quantified and compared between the two cohorts. The resulting data are shown in Figure 3B. At position 2, glutamic acid was identified in 17% of MHC class I-bound nonamers from patients with PsA, contrasting with 2% in healthy controls.

To rule out that this observation was due to the absence of the alleles HLA-B*07:02 and HLA-B*08:01 in the control cohort, the MHC class I immunopeptidome was determined for four healthy individuals per allele. In neither allele-specific control group was a greater relative abundance of glutamic acid at position 2 in MHC class I-bound nonamers observed (Supplementary Figure S3).

### 2.5. Unsupervised Clustering of MHC Class I-Bound Nonamers Reveals a Unique Glutamic Acid-Rich Cluster in PsA

**Figure 4:**
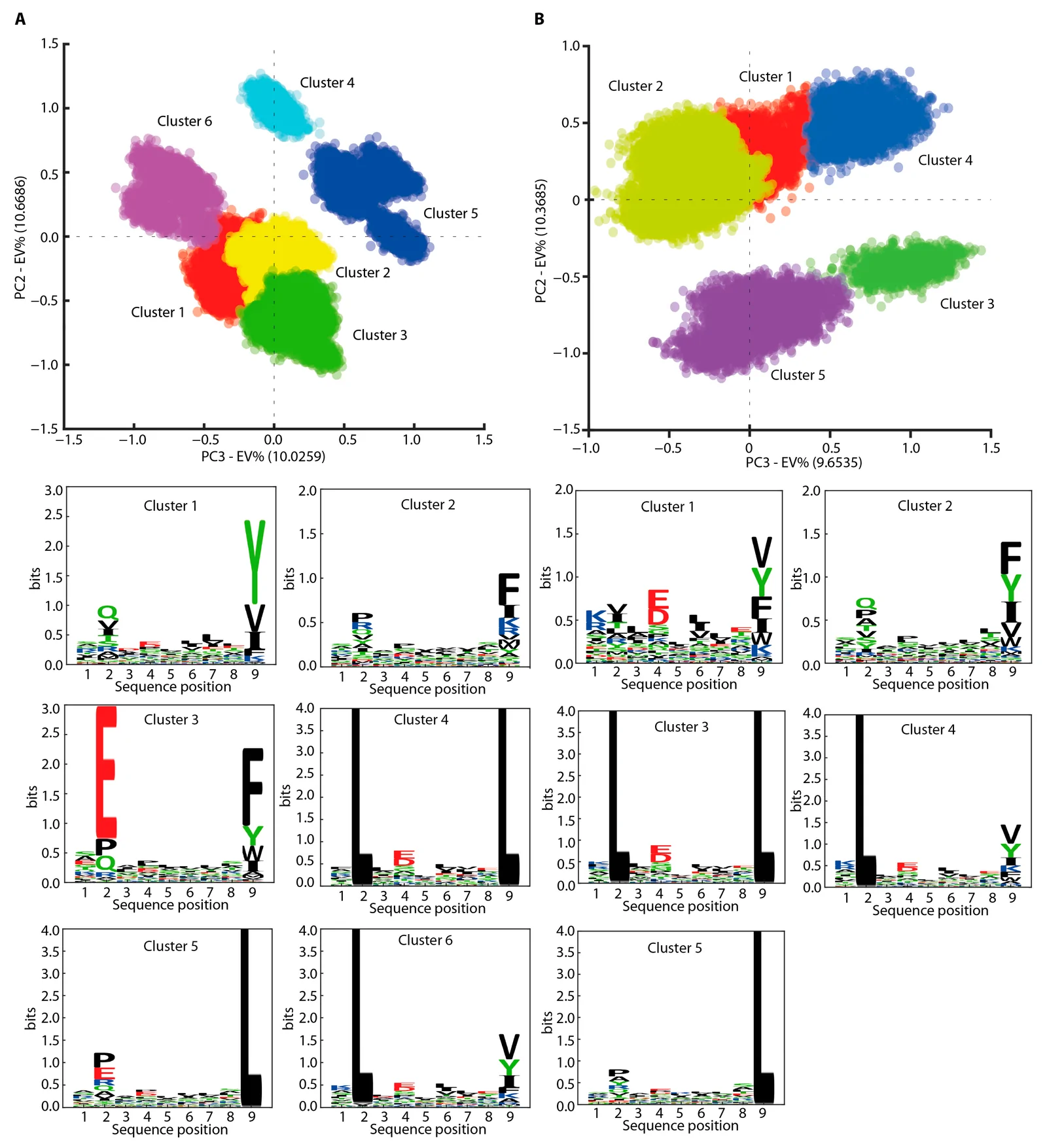
Unsupervised clustering of nonamers from MHC class I-bound peptides (**top**) and sequence logos (one letter amino acid code [24]; **bottom**) of the respective clusters in (**A**) PsA (*n* = 10) and (**B**) healthy controls (*n* = 6). The top panel shows the Principle Component Analyses (PCA) of MHC class I-bound peptides, with data points colored according to their cluster assignment. Clustering was performed using the Ward-linkage algorithm implemented in MHCpLogics, based on sequence-derived positional amino acid features that capture similarities in peptide binding motifs. The optimal number of clusters was determined using the Calinski–Harabasz criterion. Six clusters were identified in PsA patients and five clusters in healthy controls. The bottom panel depicts the sequence logos for each cluster, where letter height reflects the relative abundance of each amino acid. The sequence logos apply a simplified chemistry-based color scheme in which acidic residues appear in red, basic residues in blue, hydrophobic residues in black, and aromatic as well as small neutral residues in green. A distinct cluster with high relative abundance of glutamic acid at position 2 was identified in PsA patients (cluster 3), whereas no comparable cluster was present in healthy controls. Abbreviations: MHC, major histocompatibility complex; PCA, principal component analysis; PsA, psoriatic arthritis.

Unsupervised clustering analysis resulted in an optimal cluster number of six for the cohort of PsA patients and five for the cohort of healthy controls. The data underwent deconvolution through principal component analysis (PCA) to enhance the visual representation. The clusters were subjected to further analysis to identify their respective sequence motifs. A higher relative abundance of glutamic acid was observed in one cluster (cluster 3) in PsA, while no such cluster was found in healthy individuals (Figure 4A,B). In addition, in another cluster (cluster 5), a somewhat greater relative abundance of glutamic acid at position 2 was observed in PsA patients (Figure 4A).

### 2.6. Unique Peptide Motif from Heterogeneous Nuclear Ribonucleoprotein A1 in MHC Class I Immunopeptidome of PsA Patients

We screened the MHC class I immunopeptidomes of PsA patients and healthy individuals for the presence of peptides from putative autoantigens reported in PsA [2,7,8]. Only peptides derived from heterogeneous nuclear ribonucleoprotein A1 (hnRNP A1) were present in each PsA patient and healthy control. Peptides of other putative autoantigens [2,7,8] were less abundant or not identified in MHC class I immunopeptidomes of PsA patients and healthy controls in this study (Table 1).

**Figure 5:**
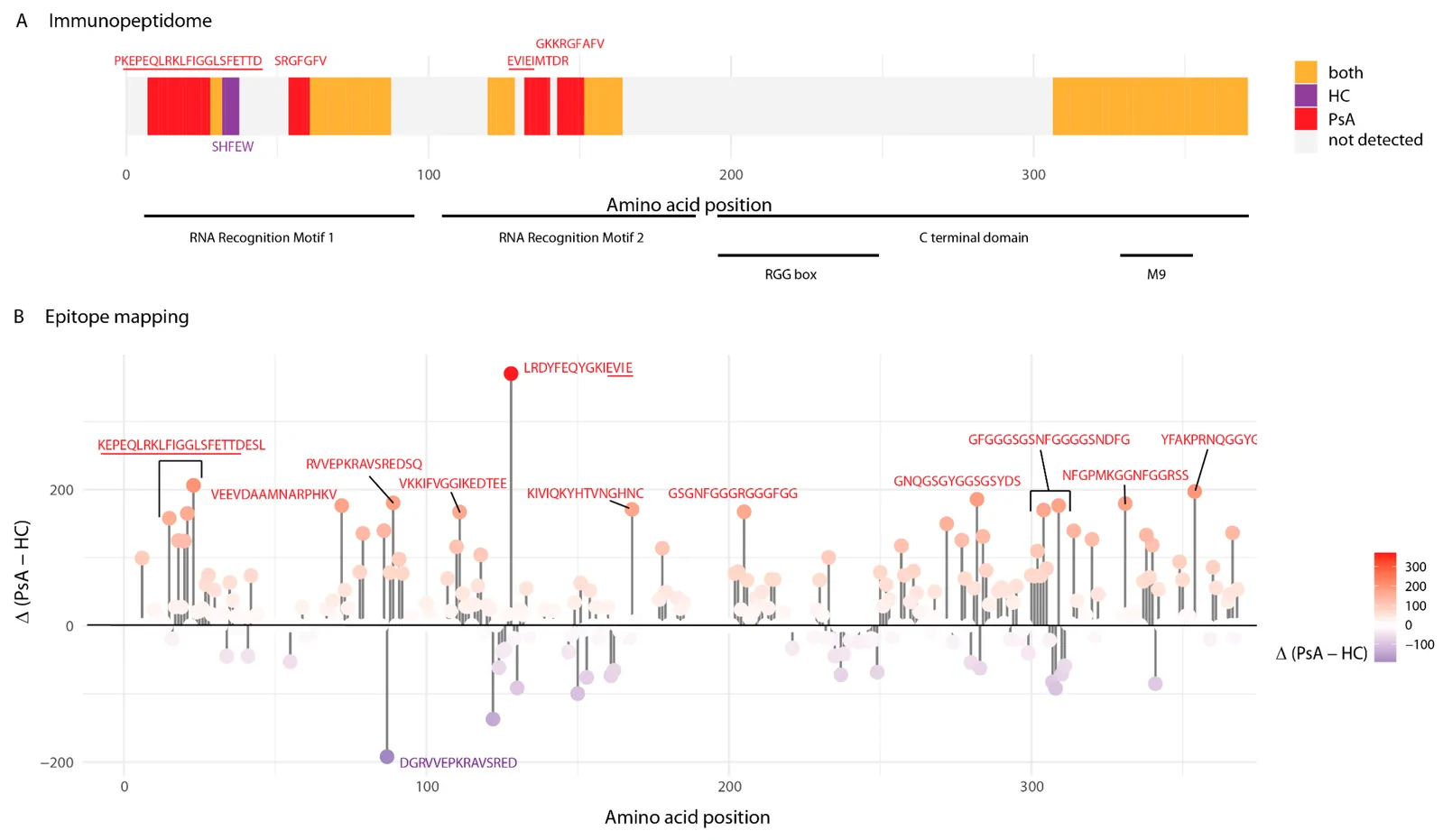
Integration of MHC class I immunopeptidome profiling and epitope mapping of heterogeneous nuclear ribonucleoprotein A1 (hnRNP A1). (**A**) Schematic representation of the hnRNP A1 isoform A1-B (372 amino acids) summarizing peptides identified in the MHC class I immunopeptidome analysis. Peptides detected in both PsA patients (*n* = 10) and healthy controls (*n* = 6) are shown in orange, PsA-exclusive peptides in red, and the single HC-exclusive peptide in lavender. Amino acid regions not presented in the MHC class I immunopeptidome are indicated in grey. (**B**) Epitope mapping of full-length hnRNP A1 using sera from five PsA patients and five healthy controls revealed overall higher fluorescence intensities in PsA. Delta-intensity values were calculated as the difference between mean fluorescence intensities of PsA and healthy control sera for each peptide spot. Delta-intensity analysis (threshold ±150 fluorescence units) identified eleven PsA-associated epitopes (red) and one HC-associated epitope (lavender). Amino acid sequences detected in both the immunopeptidome and the epitope mapping are underlined, including a PsA-specific epitope overlapping the PEQLRKLFI region. Abbreviations: HC, healthy control; hnRNP A1, heterogeneous nuclear ribonucleoprotein A1; MHC, major histocompatibility complex; PsA, psoriatic arthritis.

Among the identified MHC class I-bound hnRNP A1 peptides, 22 were found both in PsA patients and healthy controls. However, a larger proportion of hnRNP A1 peptides (*n* = 20 peptides) was exclusively present in PsA patients compared with healthy individuals (*n* = 8 peptides) (Supplementary Figure S4A). Further analysis revealed two sequence logos in the PsA cohort and one in healthy controls. In one of the two sequence logos in PsA, we again identified a greater relative abundance of glutamic acid at position 2. Moreover, the glutamic acid (E, one letter amino acid code [23]) containing PEQLRKLFI motif was identified as distinctive peptide motif in this logo of PsA patients (Supplementary Figure S4B). The PEQLRKLFI peptide sequence is located in the RNA recognition motif (RRM)1 domain of hnRNP A1 (Figure 5A). Besides the core PEQLRKLFI sequence, three short peptide motifs were detected exclusively in PsA patients, whereas one short motif was found only in healthy controls (Figure 5A). To further substantiate the immunological relevance of this peptide region, the dataset was complemented by an epitope mapping of the human hnRNP A1 protein (isoform A1-B, 372 amino acids) using sera from five PsA patients and five healthy controls. The epitope mapping revealed an overall higher fluorescence intensity in PsA patient sera compared with healthy controls (Figure 5B). By calculating the fluorescence intensity delta between PsA patients and healthy individuals, 11 potential epitopes enriched in PsA and one epitope enriched in healthy controls were identified. Notably, the amino acid region surrounding the PEQLRKLFI peptide corresponded to one of the PsA-associated epitopes.

## 3. Discussion

To our knowledge, this study demonstrates for the first time increased MHC class I expression on circulating immune cells and skewing of MHC class I-bound peptides from different intracellular compartments, including presentation of a unique peptide motif from a putative autoantigen in PsA.

Using flow cytometry analysis, we found increased MHC class I expression on circulating CD4+ and CD8+ T cells, B cells, monocytes and neutrophils in PsA. MHC class I expression correlated between different immune cell populations and between MHC class I expression on monocytes and disease activity as determined by DAS28-CRP. All patients were under treatment with either bDMARDs or tsDMARDs, having low cutaneous disease activity and overall moderate arthritis disease activity. BDMARDs, particularly those inhibiting the interleukin (IL)-23/IL-17 axis, ameliorate cutaneous disease more effectively than arthritis in PsA [25]. MHC class I expression is experimentally upregulated in the presence of the pro-inflammatory cytokines TNF-α and interferon (IFN)-γ, an effect which is inhibited by anti-TNF-α antibodies in a dose-dependent manner [26]. Thus, both disease activity and immunosuppressive therapy can affect MHC class I expression [26], with the findings of our study suggesting that MHC class I expression on circulating immune cells remained increased due to incomplete control of pro-inflammatory cytokine effects despite immunosuppressive therapy with otherwise clinically tolerated low to moderate disease activity.

Albeit a small cohort, PsA-associated HLA-B*08:01 and HLA-B*27:02 alleles [3] were found in patients with PsA, but not in healthy controls. HLA-C*06:02 associated with PsO but not PsA [1,2,3] was accordingly found in a similar proportion of patients with PsA and healthy controls. Given its strong MHC class I association suggestive of a role of MHC class I-mediated antigen presentation in driving CD8+ T cell-mediated PsA immunopathology [1,2], we analyzed the MHC class I immunopeptidome of PsA patients and healthy controls. The identified peptides were mostly 9 amino acids in length (i.e., nonamers), which is typical for peptides presented via MHC class I for recognition by CD8+ T cells [9,10]. Consistent with prior analyses of tissue-specific MHC class I peptide binding, the data showed allele-dependent variation in predicted strong and weak binders, and GO enrichment indicated diverse intracellular source proteins [10].

Among the identified MHC class I-bound nonamers, the greater relative abundance of glutamic acid at position 2 was remarkable in PsA. This finding was further corroborated by unsupervised clustering of nonamers, which identified a PsA-specific cluster with increased glutamic acid abundance at position 2 that was absent in healthy controls. Position 2 harbors one of the two primary anchor residues of MHC class I-bound peptides, engaging pocket B of the peptide-binding cleft [27]. Molecules of the HLA-B*41 allotypic family [28] and HLA-B*44 supertype comprising HLA-B*18, HLA-B*40, HLA-B*44 and HLA-B*45 [29] preferentially bind peptides with glutamic acid at this position. In our cohort, only one patient carried HLA-B*18:01, making a major contribution of these allele families to the higher glutamic acid abundance at position 2 unlikely. Additionally, we could rule out that the higher frequency of the alleles HLA-B*07:02 and HLA-B*08:01 in the PsA cohort corresponds to the higher abundance of glutamic acid at position 2. Accordingly, it is likely that the increased glutamic acid abundance at position 2 reflects disease-associated processes rather than the underlying HLA allele composition. In line with this assumption, proinflammatory cytokines have been shown to alter immunopeptidome composition in a lung cancer cell line [30]. In that model, TNF-α and IFN-γ increased MHC class I expression and changed the repertoire of MHC class I-presented peptides by skewing it towards a greater relative abundance of glutamic acid at position 2 [30]. Moreover, in melanoma cell lines and myeloma cells, CDK4/6 inhibition and an immunoproteasome activator, respectively, have been shown to alter the MHC class I immunopeptidome [31,32]. To date, no data are available on how bDMARDs or tsDMARDs influence the MHC class I immunopeptidome in autoimmune diseases.

Given reports on CD8+ T cells and/or antibodies with specificity for melano- and keratinocyte-derived autoantigens in PsA [2,7,8], we screened MHC class I immunopeptidomes of circulating immune cells from PsA patients and healthy individuals for the presence of peptides derived from those putative autoantigens. Notably, neither melanocyte-derived disintegrin-like and metalloprotease-domain-containing thrombospondin type 1 motif-like 5 (ADAMTSL5)—nor keratinocyte-derived phospholipase A2 group IVD (PLA2G4D) peptides were detected among circulating MHC class I-bound peptides, even though CD8+ T cells specific for both antigens have been isolated from psoriatic lesions and shown to produce IL-17 in PsA patients, suggestive of in situ T cell-mediated autoimmune inflammation in psoriatic disease [33,34,35]. hnRNP A1-derived peptides were identified in the MHC class I immunopeptidomes of all PsA patients and healthy individuals in this study. However, PsA patients had a higher prevalence of unique MHC class I-bound hnRNP A1 peptides, including the glutamic acid-containing PEQLRKLFI motif found exclusively in PsA. To further substantiate the immunological relevance of the hnRNP A1-derived peptides identified in the MHC class I immunopeptidome, we performed an autoantibody-based epitope mapping. Although based on humoral reactivity, epitope mapping can highlight immunologically active regions that may also be targeted by autoreactive CD8+ T cells when they overlap with naturally presented MHC class I peptides. Notably, two hnRNP A1 sequences were identified by both approaches, including one harboring the PEQLRKLFI core motif. Taken together, these findings suggest that hnRNP A1 regions detected through both techniques may represent immunologically meaningful targets in PsA.

Limitations of our study include the requirement for large cell numbers to achieve sufficient MS-based detection of MHC class I-bound peptides, which may result in missing low-abundance peptides [9]. Autoantibodies against hnRNP A1 [8,36,37] and hnRNP A1-reactive IFN-γ-secreting T cells [36] have been reported in 20–40% of PsO patients and even in healthy controls, which warrants further investigation in PsA. While circulating immune cells contain a large complement of inflammatory cells [38], these may not be representative of site-specific APC [10]. Thus, the immunogenicity of peptides [11,12] identified in our study needs to be further investigated using functional tests on T cells from peripheral blood, skin lesions, or synovial fluid. Notably, our findings are supported by an immunopeptidome analysis of skin lesions from HLA-C*06:02-positive psoriasis patients, in which a Gibbs cluster analysis likewise revealed a cluster characterized by a high abundance of glutamic acid at position 2 [39]. Nevertheless, the small cohort size in the immunopeptidome analysis limits statistical power and reduces the ability to detect rare peptides and subtle immunological differences. Due to the small sample size, our analyses could not reflect disease domains and clinical phenotypes in PsA patients. All PsA patients and healthy controls are white and of Caucasian ancestry to limit ethnicity-related bias. Therefore, the generalizability of our findings to broader PsA populations is limited and the data require confirmation in larger, clinically and ethnically heterogenous cohorts.

## 4. Methods

A full method description is included in the Supplementary Information. In brief, the study was approved by the local ethics committee (AZ 22-165) and performed in accordance with the Helsinki Declaration of 1975. All participants provided informed consent. Patients fulfilled the Classification Criteria for Psoriatic Arthritis (CASPAR) [40]. Participants were recruited between February 2023 and September 2024. Demographic data, clinical features including tender and swollen joint counts, and disease activity scores were recorded. PsA domains were classified, with cutaneous involvement assessed using the Psoriasis Area and Severity Index (PASI) and affected Body Surface Area (BSA). Nail psoriasis was identified clinically by presence of nail bed and/or nail matrix involvement, e.g., salmon patch, pitting, hyperkeratosis and/or distal onycholysis. Arthritis was evaluated using the Disease Activity in Psoriatic Arthritis (DAPSA) score and Disease Activity Score 28-C-reactive protein (DAS28-CRP). Diagnosis of enthesitis was based on clinical, ultrasound, and magnetic resonance imaging (MRI) findings. Axial disease was diagnosed based on radiographic or MRI evidence, and the Bath Ankylosing Spondylitis Disease Activity Index (BASDAI) was assessed. Dactylitis was confirmed clinically and by ultrasound.

For flow-cytometry-based characterization of MHC class I expression on peripheral blood cells and LC-MS/MS-based MHC class I immunopeptidome analysis, EDTA blood was collected from study participants. Whole-blood-derived cells were stained with fluorescence-labeled antibodies for different markers and MHC class I molecules for flow cytometry analysis and analyzed using a BD FACS Canto II flow cytometer (Heidelberg, Germany). MHC class I expression was quantified using Quantum Simply Cellular (QSC) beads (Bio-RAD, Feldkirchen, Germany) stained with anti-MHC class I antibodies (Biolegend, Koblenz, Germany).

HLA genotyping was performed at the HLA-laboratory of the Stefan Morsch Foundation (Birkenfeld, Germany). For analysis of MHC class I immunopeptidomes, MHC class I molecules from peripheral blood mononuclear cell (PBMC) lysates were isolated using pan-MHC class I-specific W6/32 antibodies (clone HB-95; ATCC, Wesel, Germany) and purified through affinity chromatography. MHC class I-bound peptides were separated by acid elution. The presence of MHC class I molecules was verified by Western blotting (Supplementary Figure S1). MHC class I eluates were purified on C18 columns and finally analyzed with LC–MS/MS (timsTOF fleX HT; Bruker, Bremen, Germany) in Thunder DDA-PASEF mode [41]. Peptides were identified with FragPipe (v22.0) using nonspecific HLA workflow [24,42,43,44]. Peptides with 7–25 amino acids were analyzed, considering modifications such as cysteinylation and validated using MSBooster (v1.2.2) and Percolator (v 3.6.5). MhcVizPipe utilizing NetMHCpan4.0 tool was used to determine the quality of the analyzed samples and binding prediction of peptides to MHC class I alleles (Supplementary Figure S2) [22,45]. MHCplogics was applied for cluster analysis and motif evaluation [46]. Specific peptide motifs were identified using GibbsCluster 2.0, and protein structures were visualized using iCn3D (v3.47.1) and AlphaFold (v3.0.3) [47,48]. Data are available via ProteomeXchange [49] with the identifier PXD062985.

Moreover, an epitope mapping (PEPperMAP^®^, PEPperPRINT, Heidelberg, Germany) against the human hnRNP A1 isoform A1-B (372 amino acids) was performed from sera from five PsA patients and 5 healthy controls.

## 5. Conclusions

In conclusion, analysis of circulating immune cells from PsA patients in this study showed increased MHC class I expression and perturbation with skewing of the MHC class I immunopeptidome towards greater relative abundance of glutamic acid at position 2 found in nonamers and presentation of a unique peptide motif from hnRNP A1, likely due to pro-inflammatory cytokine effects in the presence of incomplete disease control or another yet unidentified factor in PsA.

## Figures and Tables

**Table 1:** Number and percentage of individuals in whom peptides from putative autoantigens [2,7,8] were identified as source proteins of MHC class I-bound peptides in PsA patients (*n* = 10) and healthy controls (*n* = 6). Peptides from Heterogeneous nuclear ribonucleoprotein A1 (hnRNP A1) were identified in all PsA patients and healthy controls. Abbreviations: MHC, major histocompatibility complex; PsA, psoriatic arthritis.

	Psoriatic Arthritis (*n* = 10)	Healthy Control (*n* = 6)
Source Protein	Number of Individuals Within Each Group Displaying Peptides of the Respective Source Protein, *n* (%)	
Cathelicidin antimicrobial peptide (LL37)	1 (10%)	0 (0%)
Heterogeneous nuclear ribonucleoprotein A1 (hnRNP A1)	10 (100%)	6 (100%)
Keratin, type I cytoskeletal 17 (K1C17)	5 (50%)	6 (100%)
Disintegrin-like and metalloprotease domain containing thrombospondin type 1 motif-like 5 (ADAMTSL5)	0 (0%)	0 (0%)
Phospholipase A2 group IVD (PLA2G4D)	0 (0%)	0 (0%)

## Data Availability

Immunopeptidome data are available via ProteomeXchange with the identifier PXD062985. Further data will be provided upon reasonable request.

## Supplementary Materials

The following supporting information can be downloaded at: https://www.mdpi.com/article/10.3390/ijms27167317/s1. References [50,51] were cited in the Supplementary Materials.
