# Supplementary material for: Skewed MHC Class I Peptide Presentation in Psoriatic Arthritis
Source: Int J Mol Sci. 2026 Aug 16;27(16):7317. doi: 10.3390/ijms27167317 (PMC13513872; doi:10.3390/ijms27167317)
Supplement: Supplementary file 1 [file ijms-27-07317-s001.zip › ijms-4327195-supplementary.pdf]

Supplementary Methods

Ethical approval and study design

Ethical approval was obtained from the local ethics committee (AZ 22-165) in accordance with the Helsinki Declaration of 1975. All patients gave informed consent to participate in the study. In the analysis of the MHC class I cell surface expression 14 patients with psoriatic arthritis and 12 healthy controls were included. In addition, a total of 10 patients with psoriatic arthritis and 6 healthy controls were enrolled for analysis of the MHCI immunopeptidome. All enrolled PsA patients and healthy controls were white and of Caucasian ancestry. Diagnosis of psoriatic arthritis was set according to Classification Criteria for Psoriatic Arthritis (CASPAR) criteria.[1] Study participants were recruited between 02nd February 2023 and 06th September 2024. Inclusion criteria for patients with PsA were that patients were aged > 18 years and that they fulfill the CASPAR criteria. Patients in pregnancy and lactation were excluded from study participation. Healthy controls were enrolled based on the information that they do not suffer from an acute or chronic illness, that they are currently symptom-free and that there is no known family history of psoriatic arthritis.

Demographic parameters, clinical data including tender and swollen joint counts, and disease activity scores were assessed. Based on the history of disease, the patients were grouped for the presence of psoriatic arthritis domains. Cutaneous disease manifestations were characterized by psoriasis activity as assessed by Psoriasis Area and Severity Index (PASI) and affected body surface area (BSA). Nail psoriasis was defined by distal onycholysis, hyperkeratosis, or pitting. Arthritic disease manifestation was assessed by evaluating the Disease Activity in Psoriatic Arthritis (DAPSA) and Disease Activity Score 28-C-Reactive Protein (DAS28-CRP). The diagnosis of enthesitis was made clinically using ultrasound and MR imaging. Axial disease manifestation was diagnosed based on radiographic or MR imaging of the spine the Bath Ankylosing Spondylitis Disease Activity Index (BASDAI) assessed. Dactylitis was diagnosed clinically and confirmed by ultrasound.

Determination of HLA genotype by DNA sequencing

Two milliliters of whole ethylenediaminetetraacetic acid (EDTA)-blood were sampled in Sarstedt tubes (#01.1605, Sarstedt, Germany), frozen and stored at -80 °C until shipment on dry ice. The genotyping was conducted by the laboratory of Dr. Marco Schäfer at the Stefan-Morsch-Stiftung, Birkenfeld, Germany.

Flow Cytometry-Based Characterization of MHCI Expression on human Immune Cells

Whole blood samples were collected in EDTA tubes. Subsequently, red blood cell lysis was performed using RBC Lysis Buffer (#420302, BioLegend, USA) for 12 minutes at room temperature. The samples were then mixed with Phosphate Buffered Saline (PBS) (#L0615-500, Biowest, France) and 0.5% Bovine Serum Albumin (BSA) (#P06-1391500, PAN-Biotech, Germany) and centrifuged at 650 x g for 10 minutes at room temperature. The pellet was resuspended, and this washing step was repeated twice. After the final wash, the cell pellet was resuspended in PBS-0.5% BSA. To reduce nonspecific binding, Human TruStain FcX (#422302, BioLegend, USA) and True-Stain Monocyte Blocker (#26102, BioLegend, USA) were added. The samples were vortexed and incubated at room temperature for 10 minutes. Subsequently, the cell suspension was added to the antibodies listed in **Supplementary Table S4** and incubated for 20 minutes in the dark. Following incubation, PBS-0.5% BSA was added to each tube, and the cells were washed by centrifugation at 650 x g for 10 minutes at room temperature. The supernatant was carefully aspirated, and the pellet was resuspended in PBS-0.5% BSA.

For quantification of MHC I expression, Quantum Simply Cellular (QSC) beads (#815B, Bangs Laboratories, USA) were used. Beads were stained with anti-MHC I (PE) antibodies by incubating in the dark at room temperature for 30 minutes. Beads were then washed twice in PBS-0.5% BSA, centrifuging at 2,500 x g for 5 minutes at room temperature, and carefully removing the supernatant. Finally, beads were resuspended in PBS-0.5% BSA.

The samples were transferred to BD FACS Canto II tubes for measurement. The samples were analyzed using the BD FACS Canto II flow cytometer (Heidelberg, Germany). Data analysis

was performed using the FlowJo™ software v10 (BD Biosciences, USA). Doublets were excluded based on forward scatter height (FSC-H) and forward scatter area (FSC-A). Lymphocytes, monocytes, and neutrophils were identified based on their size (FSC-A) and granularity (side scatter area, SSC-A). Lymphocytes were further classified as CD3+ and subsequently subdivided according to CD4 and CD8 expression. B cells were defined as CD3- and CD19+ lymphocytes. Neutrophils were further categorized by CD16 positivity, while monocytes were identified based on CD14 positivity. Finally, the frequency of MHC I+ cells was determined through gating.

Isolation of peripheral mononuclear cells derived from human blood

EDTA-blood (25 ml) drawn from patients with psoriatic arthritis and healthy controls was diluted in a 1:1 ratio with PBS (#L0615-500, Biowest, France). Then, 25 mL of BiColl solution (#BS.L6115, Bio & Sell, Germany) were carefully covered with the diluted EDTA-blood. Subsequently, the blood was subjected to centrifugation at 800 x g for 25 minutes without a brake and with low acceleration at room temperature. The plasma layer that formed during centrifugation was discarded, and the cloudy intermediate layer, which contained the peripheral blood mononuclear cells, was carefully isolated using a pipette. Subsequently, the cells were washed twice with cold PBS at 650 x g for 7 minutes at 4 °C. The cells were washed once more with cold PBS at 650 x g for 7 minutes at 4 °C. Subsequently, the supernatant was removed. The cells were cryopreserved in liquid nitrogen and stored at -80 °C.

Cultivation of murine hybridoma cells of the HB-95 clone

For the production of the monoclonal α-MHC class I antibody W6/32, the murine hybridoma cells of the HB-95 clone (#HB-95, ATCC, USA) were used. The hybridoma cells were cultured in hybridoma complete medium composed of RPMI 1640 (#BS.FG1215, Bio & Sell, Germany), 10% Immunoglobulin G (IgG)-depleted fetal calf serum (FCS) (#BS.370845D, Bio & Sell, Germany) and 1% Penicillin-Streptomycin (#L-0022-100, Biowest, France) at 5% CO_2, 37 °C. To stimulate antibody production through stress induction, the cells were transferred to a

serum-depleted permanent culture following sufficient expansion. This was achieved by diluting the cell suspension 1:5 with serum-free hybridoma complete medium. Subsequently, the cells were incubated for a period of three weeks until they could be harvested.

To generate the IgG-depleted FCS, it was sterilized by filtration and deactivated at 56 °C for one hour. To eliminate the bovine IgG, the FCS (#BS.370845D, Bio&Sell, Germany) was purified through affinity chromatography using Äkta Start (Cytiva, USA). The HiTrap Protein G HP column (#17040501, Cytiva, USA) was initially washed with phosphate buffer (20 mM Na2HPO4 (#4984.1, Carl ROTH, Germany), 20 mM NaH2PO4 (#K300.1, Carl ROTH, Germany), pH=7.0), and subsequently the FCS was passed over the column twice under sterile conditions and used for medium.

Purification of the Monoclonal α-MHC Class I Antibody W6/32

To purify the monoclonal α-MHC Class I Antibody W6/32, the hybridoma cell suspension was centrifuged at maximum speed for 10 minutes at 4 °C. Subsequently, the supernatant was sterile filtered and stored at 4 °C in the dark until purification. The W6/32 antibodies were purified using Äkta Start (Cytiva, USA). The HiTrap Protein G HP column (#17040501, Cytiva, USA) was initially washed with phosphate buffer as described above, and then the cell culture supernatant was passed over the column twice under sterile conditions. The antibody was eluted with 0.1 M glycine buffer (#G8898.500g, Sigma-Aldrich, USA) at pH=2.7 and subsequently neutralized with 1 M TRIS/HCl at a pH of 11.0 (TRIS: #37190.02, SERVA, Germany; HCl: #4625.2, Carl ROTH, Germany). The eluted antibody was buffered twice overnight at 4 °C against PBS using dialysis tubing (#0653.1, Carl ROTH, Germany) and concentrated using polyethylenglycol (#33137.01, SERVA, Germany). The purified antibody was then frozen at -80 °C for long-term storage.

Affinity chromatography for MHC class I complex purification

MHC class I complexes were purified from isolated human peripheral blood mononuclear cells using affinity chromatography. The isolated human peripheral blood mononuclear cells were

lysed by adding 2 x IP Lysis Buffer (16.3 mM CHAPS (#C-3023, Sigma-Aldrich, USA), 1 x Halt Proteinase Inhibitor Cocktail (#1862209, Thermo Scientific, USA), 1 mM Phenylmethylsulfonyl fluoride (#A0999.0005, AppliChem, Germany)). The lysis was conducted at 4 °C for one hour with constant agitation. Subsequently, the lysed cells were subjected to centrifugation at 13,000 rpm for 20 minutes at 4 °C. For affinity chromatography, 5 mg of the W6/32 antibodies were immobilized to a Sepharose matrix using AminoLink Plus Immobilization columns (#44894, Thermo Scientific, Germany) according to the manufacturer's protocol (pH=7.2). Next, the supernatant of the cell lysate was added to the AminoLink Plus Immobilization columns. The incubation process was conducted overnight at 4 °C with rotation. For purification all centrifugations were conducted at 1,000 x g for one minute at room temperature, and all flow-through fractions were collected for subsequent western blot analysis. Subsequent to centrifuging the column, the following washing procedures were carried out: 10 washes with IP Wash Buffer A (WA; 150 mM NaCl (#146994.1214, AppliChem, Germany), 20 mM TRIS, pH=8.0), then 10 washes with IP Wash Buffer B (WB; 400 mM NaCl, 20 mM TRIS, pH=8.0), followed by again 10 washes with IP Wash Buffer A, and finally 10 washes with 4 mL IP Wash Buffer C (WC; 20 mM TRIS, pH=8.0). Following the washing step, elution E1 and E2 was performed twice by adding IP Elution Buffer (1% trifluoric acid (TFA) (#1.08178.0050, Sigma-Aldrich, USA)), incubating for 5 minutes, and centrifuging the column. The second elution (E2) was followed by a post-elution washing with PBS (WP). The flow-through contained purified MHC class I.

Western blot analysis to confirm MHC class I purification

To estimate the efficacy of MHC class I purification, Western blot analysis was performed for the cell lysate (L), flow through (FT), elution 1 and 2 (E1, E2) beads (B) and all washing fractions (WA, WB, WC, WP). These listed samples were combined with 4 x Laemmli buffer (#1610747, Bio-Rad Laboratories, USA) containing 5% β-mercaptoethanol (#M3148, Sigma-Aldrich, USA). Subsequently, the samples were heated at 95 °C for 5 minutes. All samples were loaded on the Criterion TGX Precast Gels 4-20% (#5671094, Bio-Rad Laboratories, USA)

without dilution except the flow-through, which was diluted 1:3 with PBS prior to the addition of Laemmli buffer. Precision Plus Protein Dual Color Standard (#1610374, Bio-Rad Laboratories, USA) was used as a marker. The electrophoresis was conducted for a period of 40 minutes at a constant voltage of 200 V and a maximum current of 300 mA using a SDS Gel chamber (Bio-Rad Laboratories, USA). Next, proteins were transferred to a methanol-activated polyvinylidene fluoride (PVDF) membrane (#1620177, Bio-Rad Laboratories, USA) using the Turboblotter (Bio-Rad Laboratories, USA) for a period of 25 minutes at a current of 2.5 A and a voltage of 25 V. Subsequently, the membrane was blocked in Blocking Buffer (150 mM NaCl, 50 mM TRIS, 0.05% Tween 20 (#2.42312.1611, AppliChem, USA), 5% BSA, pH=7.5) for one hour at room temperature with gentle rocking. The blot was incubated with the primary antibody, which was MHC Class I monoclonal antibody (#88274S, CellSignaling Technology, USA) diluted 1:100 in Blocking Buffer, for a period of overnight at 4 °C. On the subsequent day, the membrane was washed three times in Washing Buffer (150 mM NaCl, 50 mM TRIS, 0.05% Tween 20, pH=7.5), followed by incubation with secondary antibody, peroxidase-conjugated goat anti-mouse IgG (#15-035-003, Jackson-ImmunoResearch Laboratories, USA), which was diluted 1:20,000 in Blocking Buffer, for 60 minutes at room temperature and gentle rocking. Subsequently, the blot was washed five times in Washing Buffer. Finally, MHC class I complexes were detected using Supersignal West Pico Plus (#34580, Thermo Scientific, USA) and analyzed with the Fusion detection device (Vilber, France).

Isolation of MHC class I-bound peptides

C18 EvoTip disposable trap columns (EV20128, Evosep, Denmark) were used for the isolation of the peptides presented via MHC I. C18 column was pre-activated by adding 1 mL C18 Activation Buffer (15.3 mM acetonitrile (ACN) (#271004-1L, Sigma-Aldrich, USA), 13.1 µM TFA). The column was then centrifuged at 300 x g for 1 minute at room temperature. This step was repeated. After that, the column was washed twice using 1 mL C18 Desalting Buffer (13.1 µM TFA) by centrifugation as mentioned before. At this point, the column was ready to be loaded with eluted MHC I. The column was centrifuged, and this step was repeated until the

whole eluate was loaded. To clean the loaded peptides, the column was washed three times using 1 mL C18 Desalting Buffer and the peptides were finally eluted by adding 1 mL C18 Elution Buffer (5.4 mM ACN, 13.1μM TFA) and centrifugation at 300 x g for 1 minute at room temperature. The flow through contained purified peptides. Elution was performed three times, and the eluate was stored at -80 °C until further use.

Liquid chromatography coupled mass spectrometry

Chromatography was performed on an EvoSep One using a C18 Performance column (EV1137, Evosep, Denmark) in combination with the extended 15 SPD method (88 min gradient, 220 nl/min). Mobile phases A and B were water with 26.5 μM formic acid (Baker Thermo Fischer, Waltham, USA) and 19.1 μM ACN, respectively. The liquid chromatography system was coupled to timsTOF fleX HT mass spectrometer (Bruker, USA) via a CaptiveSpray nano-electrospray ion source (Bruker, USA). The mass spectrometer was operated in Thunder-DDA-PASEF mode. The corresponding LC-MS/MS method, initially described by Gomez-Zepeda et al.[2], featured a HLA peptide ligand-tailored isolation polygon, a 300 ms TIMS ramp and three MS2 frames/cycle, one cycle overlap and utilized the high-sensitivity mode.

Proteomics database search

For the proteomics database search, FragPipe (v2.2) was used, employing the nonspecific-HLA workflow in combination with IonQuant for quantification of peptides.[3–7] Briefly, MSFragger searches for peptides of 7-25 amino acids length, assuming cysteines were not alkylated. Cysteinylation is specified as variable modification. MSFragger results are rescored with MSBooster, peptide spectrum matches are validated with Percolator.

Peptidomics data analysis using MHCVizPipe and MHCpLogics algorithms

For the examination of the predicted binding of the peptides and thus for an estimation of data quality, mass spectrometry data and genetic data on the MHC class I alleles were combined

using the MHCVizPipe algorithm.[8] The MHCVizPipe algorithm utilizes the NetMHCpan 4.0 tool, which was developed by DTU Health Tech in Denmark.[9] The analysis using the MHCVizPipe algorithm was performed for each study participant individually. Length factors (LF), calculated as the fraction of 8-14mers in the sample, and binding factors (BF), calculated as the fraction of binding peptides among 8-14mers in the sample, were utilized to evaluate the quality of the sample. For each study participant, peptides with a length between 8 and 12 amino acids were analyzed in order to ascertain their predicted binding for each MHC class I allele. The eluted ligands (EL) % rank was employed as a metric for determining which peptides exhibited binding affinity. An EL % rank of less than 0.5 indicates a peptide with a high affinity for the MHC class I allele, while a rank greater than 0.5 and less than 2 indicates a peptide with a lower affinity. Peptides with a rank greater than 2 are classified as non-binders. A color-coded heatmap was constructed for each participant, illustrating the number of peptides and their predicted binding for each MHC class I allele.

The following analyses of the peptidomics data were pursued using MHCpLogics. MHCpLogics is a tool developed by Shahbazy et al., which uses a supervised learning method leaving out the individuals' genotype.[10] MHCplogics was used for cluster analysis and motif evaluation based on data labeled as belonging to psoriatic arthritis or healthy controls. The length of the peptides, which were selected for analysis was set to 8-14 amino acids and peptides were encoded by logical-based fingerprint sequence encoding (LFSE). Subsequently, clustering was performed using Ward Linkage algorithm with the optimal cluster number calculated by Calinski–Harabasz criterion. Principle component analysis (PCA) enhanced readability by dimensional reduction. The PCA residues were color-coded according to their annotated cluster. Length distribution, sequence logos and amino acid frequencies for each position of the logo were generated for each cluster and the whole group, as well as a Venn diagram for the peptide's sequences of each group.

Autoantigens were searched by manual analysis and counted. Analysis of hnRNP A1 peptides was performed by Venn diagram, to evaluate overlaps. Sequence motifs of peptides, which were unique for one group, were created using GibbsCluster for MHC class I peptides.[11] 3D

protein structure and peptide position within was evaluated by iCn3D using AlphaFold.[12,13] The MS proteomics data have been deposited to the ProteomeXchange Consortium via the PRIDE partner repository[14] with the dataset identifier PXD062985.

PEPperMap® epitope mapping of human sera against hnRNP A1

PEPperMap® epitope mapping was performed according to the protocols of the company PEPperPrint GmbH (Heidelberg, Germany). The amino acid sequence of human hnRNPA1 (UniProt P09651) was extended at both the N- and C-terminus with neutral GSGSGSG linkers to avoid truncated peptide ends. The elongated sequence was converted into linear 15-mer peptides with an overlap of 14 amino acids resulting in a peptide library of 372 unique hnRNPA1 peptides, each printed in duplicate. The arrays were framed by control peptides. Prior to sample incubation, one microarray was pre-stained with the secondary and control antibodies to assess background interactions. The further microarrays were blocked for 30 min using Rockland MB-070 blocking buffer. Serum samples from five PsA patients and 5 healthy controls, that were also included in the MHC class I immunopeptidome analysis were diluted (1:200) and incubated on the microarrays for 16 h at 4 °C with orbital shaking at 140 rpm. Afterwards, arrays were washed three times for 10 s each with PBS (pH 7.4) containing 0.05% Tween-20. Bound human IgG was detected using goat anti-human IgG (Fc) DyLight680 (0.1 µg/ml) for 45 min at room temperature. Arrays were scanned using an Innopsys InnoScan 710-IR microarray scanner at 20 µm resolution. The DyLight680 channel (red, 680 nm) was acquired at low laser power with a gain of 50, and the DyLight800 channel (green, 800 nm) at high laser power with a gain of 10. Microarray image analysis was performed using PepSlide® Analyzer. The software algorithm extracted raw, foreground, and background fluorescence intensities for each spot. Analyses were based on the median foreground intensities of each spot. Duplicate spots were evaluated for consistency, and a maximum spot-to-spot deviation of 60% was tolerated. Using RStudio 2026.05.1, mean fluorescence intensities for PsA and HC sera were calculated and converted into Δ-values for each peptide. These Δ-values were

visualized along the hnRNP A1 sequence using a lollipop plot, with peptides showing Δ-values ≥ 150 or ≤ -150 highlighted.

Statistical analysis

Data were tested for parametric properties using the Shapiro-Wilk test. As the datasets did not meet the assumptions of normality, the antibody-binding capacity of $CD4^+$ T cells, $CD8^+$ T cells, monocytes, neutrophils, and B cells was compared using the Mann-Whitney *U* test. Correlations between MHC class I cell-surface expression on immune cell subsets and clinical parameters were assessed by calculating Spearman's rank correlation coefficient (R) together with the corresponding unadjusted p-values. Spearman's rank correlation coefficients (R) greater than 0.5 were considered indicative of a strong correlation.

Except for the GO enrichment analysis, where Benjamini–Hochberg correction was applied, no multiple-testing correction was used. All other analyses involved a limited number of predefined comparisons addressing distinct biological questions, and results are therefore reported using unadjusted p-values and interpreted descriptively. For reference, Bonferroni-adjusted significance thresholds of $p<0.0005$ can be considered.

References

[1] Taylor W, Gladman D, Helliwell P, Marchesoni A, Mease P, Mielants H. Classification criteria for psoriatic arthritis: Development of new criteria from a large international study. *Arthritis & Rheumatism* 2006 Aug;54(8):2665–73.

[2] Gomez-Zepeda D, Arnold-Schild D, Beyrle J, Declercq A, Gabriels R, Kumm E, et al. Thunder-DDA-PASEF enables high-coverage immunopeptidomics and is boosted by MS2Rescore with MS2PIP timsTOF fragmentation prediction model. *Nat Commun* 2024 Mar 13;15(1):2288.

[3] Kong AT, Leprevost FV, Avtonomov DM, Mellacheruvu D, Nesvizhskii AI. MSFragger: ultrafast and comprehensive peptide identification in mass spectrometry–based proteomics. *Nat Methods* 2017 May;14(5):513–20.

[4] Yu F, Haynes SE, Teo GC, Avtonomov DM, Polasky DA, Nesvizhskii AI. Fast Quantitative Analysis of timsTOF PASEF Data with MSFragger and IonQuant. *Molecular & Cellular Proteomics* 2020 Sep;19(9):1575–85.

[5] Yu F, Teo GC, Kong AT, Haynes SE, Avtonomov DM, Geiszler DJ, et al. Identification of modified peptides using localization-aware open search. *Nat Commun* 2020 Aug 13;11(1):4065.

[6] Yu F, Haynes SE, Nesvizhskii AI. IonQuant Enables Accurate and Sensitive Label-Free Quantification With FDR-Controlled Match-Between-Runs. *Molecular & Cellular Proteomics* 2021;20:100077.

[7] Teo GC, Polasky DA, Yu F, Nesvizhskii AI. Fast Deisotoping Algorithm and Its Implementation in the MSFragger Search Engine. *J Proteome Res* 2021 Jan 1;20(1):498–505.

[8] Kovalchik KA, Ma Q, Wessling L, Saab F, Duquette JD, Kubiniok P, et al. MhcVizPipe: A Quality Control Software for Rapid Assessment of Small- to Large-Scale Immunopeptidome Datasets. *Molecular & Cellular Proteomics* 2022 Jan;21(1):100178.

[9] Jurtz V, Paul S, Andreatta M, Marcatili P, Peters B, Nielsen M. NetMHCpan-4.0: Improved Peptide–MHC Class I Interaction Predictions Integrating Eluted Ligand and Peptide Binding Affinity Data. *The Journal of Immunology* 2017 Nov 1;199(9):3360–8.

[10] Shahbazy M, Ramarathinam SH, Li C, Illing PT, Faridi P, Croft NP, et al. MHCpLogics: an interactive machine learning-based tool for unsupervised data visualization and cluster analysis of immunopeptidomes. *Briefings in Bioinformatics* 2024 Jan 22;25(2):bbae087.

[11] Andreatta M, Alvarez B, Nielsen M. GibbsCluster: unsupervised clustering and alignment of peptide sequences. *Nucleic Acids Research* 2017 Jul 3;45(W1):W458–63.

[12] Jumper J, Evans R, Pritzel A, Green T, Figurnov M, Ronneberger O, et al. Highly accurate protein structure prediction with AlphaFold. *Nature* 2021 Aug 26;596(7873):583–9.

[13] Wang J, Youkharibache P, Zhang D, Lanczycki CJ, Geer RC, Madej T, et al. iCn3D, a web-based 3D viewer for sharing 1D/2D/3D representations of biomolecular structures. Valencia A, editor. *Bioinformatics*. 2020 Jan 1;36(1):131–5.

[14] Deutsch EW, Bandeira N, Perez-Riverol Y, *et al.* The ProteomeXchange consortium at 10 years: 2023 update. *Nucleic Acids Res* 2023 Jan 26;51(D1):D1539-D1548.

Supplement – Figures and tables

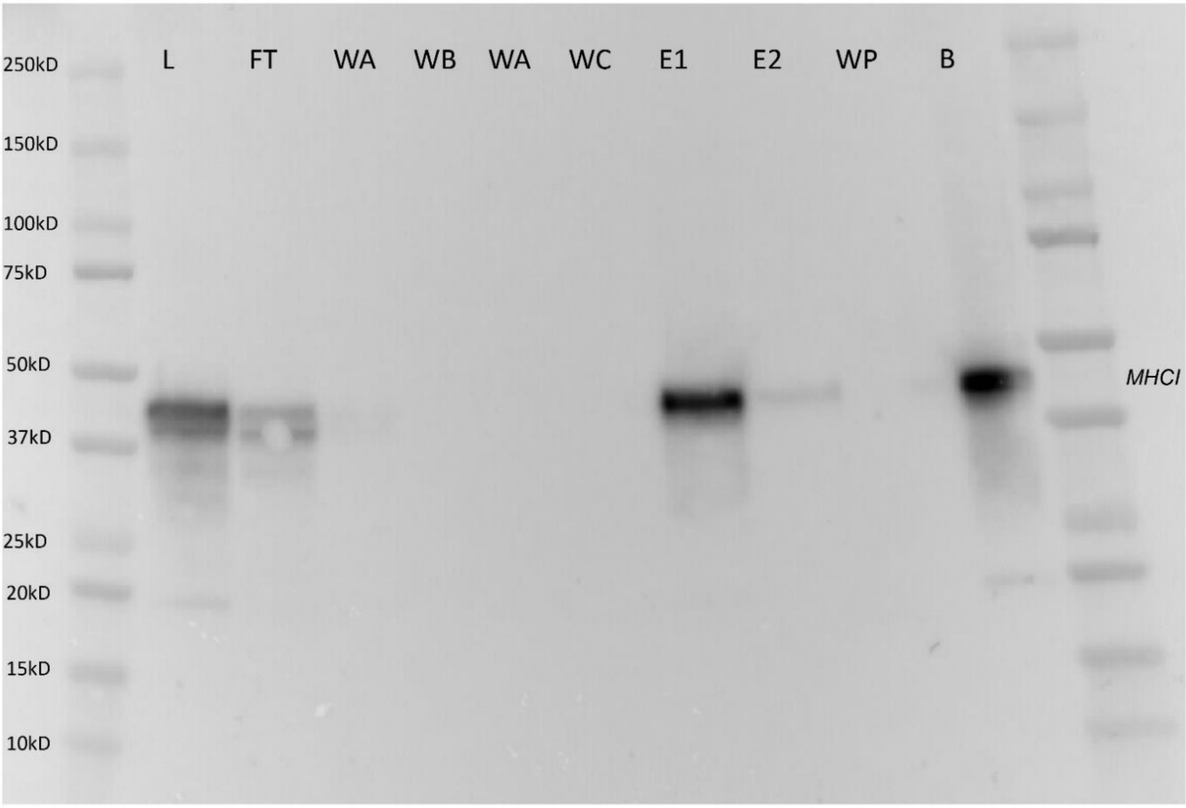

Supplementary Figure S1 Western blot analysis for MHC class I detection. Fractions of every purification step were analyzed by Western Blot: lysate (L), flow through (FT), elution 1 and 2 (E1, E2), and MHC class-covered beads (B). Washing fractions (WA, WB, WC, WP) with different buffers contained no detectable MHC class I. Western Blot analysis using mouse anti-human MHC class I antibody as primary antibody and peroxidase coupled goat anti-mouse IgG secondary antibody to evaluate efficacy of MHC class I isolation derived from PBMC of a healthy donor.

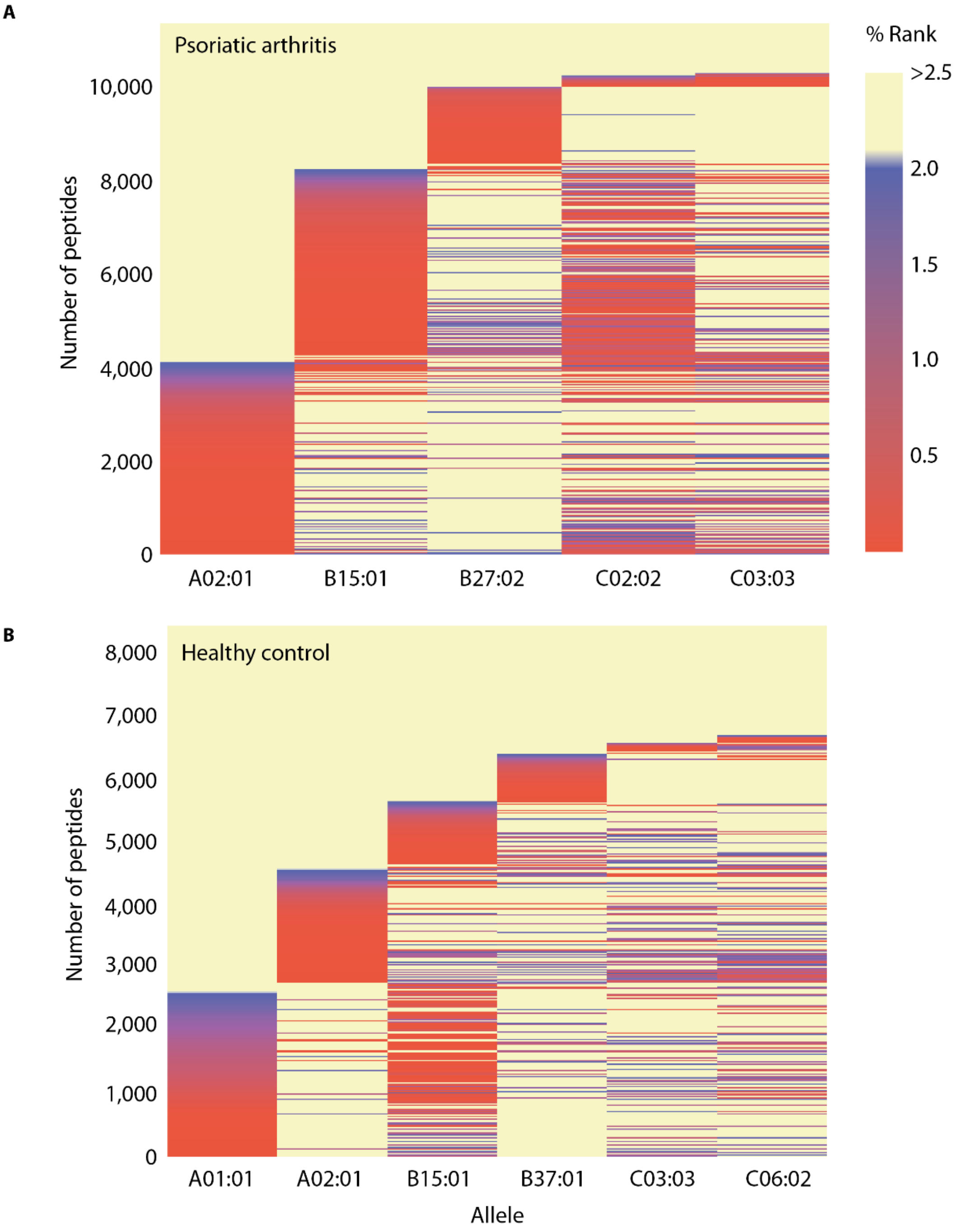

Supplementary Figure S2 MhcVizPipe application for binding prediction of peptides to MHC class I alleles. Predicted binding scores of analyzed peptides according to each allele are color-coded. Cut off values: %rank ≤0.5 strong binder, %rank ≤2 weak binder, %rank >2 non-binders. Example of MhcVizPipe binding heatmap of a sample from a patient with PsA (top) and healthy control (bottom).

	**Psoriatic arthritis (n=14)**	**Healthy controls (n=12)**
**Sex**:		
Male, n (%)	5 (35.7%)	2 (16.7%)
Female, n (%)	9 (64.3%)	10 (83.3%)
**Age**, years (mean ± SD)	49.43 ± 9.5	56.11 ± 10.2
**BMI**, kg/m^2 (mean ± SD)	29.81 ± 3.2	
**Disease duration**, months (mean ± SD)	181.2 ± 370.8	
**Disease domains**		
Psoriasis, n (%)	10 (71.4%)	
Nail psoriasis, n (%)	10 (71.4%)	
Enthesitis, n (%)	11 (78.6%)	
Peripheral arthritis, n (%)	12 (85.7%)	
Axial manifestation, n (%)	3 (21.4%)	
Dactylitis, n (%)	4 (28.6%)	
Uveitis, n (%)	0 (0.0%)	
**Disease activity scores**		
PASI (mean ± SD)	2.40 ± 3.02	
BSA (mean ± SD)	2.82 ± 13.32	
DAS28-CRP (mean ± SD)	3.12 ± 1.23	
DAPSA (mean ± SD)	18.54 ± 11.77	
BASDAI (mean ± SD)	4.72 ± 1.82	
**Therapeutic regimen**		
TNF inhibitor, n (%)	2 (14.3)	
IL-23 inhibitor, n (%)	5 (35.7)	
IL-17 inhibitor, n (%)	6 (42.9)	
JAK inhibitor, n (%)	1 (7.1)	

Supplementary Table S1. Demographic and clinical characterization of patients with PsA and healthy controls included for MHC class I cell surface expression. In 4 PsA patients both FACS and LC-MS/MS-based immunopeptidome analysis were performed. *Abbreviations: BASDAI, Bath Ankylosing Spondylitis Disease Activity Index; BMI, body mass index; BSA, body surface area; DAPSA, Disease Activity in PsA; DAS28-CRP, Disease Activity Score with 28 joints and C-reactive protein; IL, interleukin; PASI, Psoriasis Area and Severity Index.*

	**Psoriatic arthritis (n=10)**	**Healthy controls (n=6)**
**Sex**:		
Male, n (%)	5 (50.0%)	2 (33.3%)
Female, n (%)	5 (50.0%)	4 (66.6%)
**Age**, years (mean ± SD)	49.8 ± 5.2	41.7 ± 13.9
**BMI**, kg/m^2 (mean ± SD)	31.0 ± 6.0	
**Disease duration**, months (mean ± SD)	105.8 ± 140.9	
**Disease domains**		
Psoriasis, n (%)	9 (90.0%)	
Nail psoriasis, n (%)	6 (60.0%)	
Enthesitis, n (%)	6 (60.0%)	
Peripheral arthritis, n (%)	9 (90.0%)	
Axial manifestation, n (%)	3 (30.0%)	
Dactylitis, n (%)	1 (10.0%)	
Uveitis, n (%)	1 (10.0%)	
**Disease activity scores**		
PASI (mean ± SD)	2.3 ± 1.8	
BSA (mean ± SD)	2.8 ± 1.7	
DAS28-CRP (mean ± SD)	2.5 ± 0.9	
DAPSA (mean ± SD)	14.5 ± 8.7	
BASDAI (mean ± SD)	5.1 ± 2.5	
**Therapeutic regimen**		
TNF inhibitor, n (%)	3 (30%)	
IL-23 inhibitor, n (%)	2 (20%)	
IL-17 inhibitor, n (%)	4 (40%)	
JAK inhibitor, n (%)	1 (10%)	

Supplementary Table S2. Demographic and clinical characterization of patients with PsA and healthy controls included for LC-MS/MS-based MHC class I cell immunopeptidome analysis. In 4 PsA patients both FACS and LC-MS/MS-based immunopeptidome analysis were performed. *Abbreviations: BASDAI, Bath Ankylosing Spondylitis Disease Activity Index; BMI, body mass index; BSA, body surface area; DAPSA, Disease Activity in PsA; DAS28-CRP, Disease Activity Score with 28 joints and C-reactive protein; IL, interleukin; PASI, Psoriasis Area and Severity Index.*

HLA allele	Psoriatic arthritis (n=10)	Healthy controls (n=6)
**HLA-A**	n (%)	n (%)
*HLA-A*01:01*	5 (25.0%)	3 (25.0%)
*HLA-A*02:01*	7 (35.0%)	5 (41.7%)
*HLA-A*02:05*	1 (5.0%)	0 (0.0%)
*HLA-A*03:01*	3 (15.0%)	1 (8.3%)
*HLA-A*24:02*	1 (5.0%)	2 (16.7%)
*HLA-A*31:01*	1 (5.0%)	0 (0.0%)
*HLA-A*32:01*	0 (0.0%)	1 (8.3%)
*HLA-A*68:01*	1 (5.0%)	0 (0.0%)
*HLA-A*68:02*	1 (5.0%)	0 (0.0%)
**HLA-B**		
*HLA-B*07:02*	5 (25.0%)	0 (0.0%)
*HLA-B*08:01*	5 (25.0%)	0 (0.0%)
*HLA-B*13:02*	1 (5.0%)	1 (8.3%)
*HLA-B*14:02*	1 (5.0%)	1 (8.3%)
*HLA-B*15:01*	2 (10.0%)	3 (25.0%)
*HLA-B*18:01*	1 (5.0%)	0 (0.0%)
*HLA-B*27:02*	2 (10.0%)	0 (0.0%)
*HLA-B*27:05*	0 (0.0%)	1 (8.3%)
*HLA-B*35:01*	1 (5.0%)	0 (0.0%)
*HLA-B*37:01*	0 (0.0%)	1 (8.3%)
*HLA-B*44:03*	0 (0.0%)	1 (8.3%)
*HLA-B*44:05*	0 (0.0%)	1 (8.3%)
*HLA-B*50:01*	2 (10.0%)	0 (0.0%)
*HLA-B*51:01*	0 (0.0%)	2 (16.7%)
*HLA-B*57:01*	0 (0.0%)	1 (8.3%)
**HLA-C**		
*HLA-C*01:02*	0 (0.0%)	1 (8.3%)
*HLA-C*02:02*	2 (10.0%)	2 (16.7)
*HLA-C*03:03*	1 (5.0%)	3 (25.0%)
*HLA-C*05:01*	0 (0.0%)	1 (8.3%)
*HLA-C*06:02*	4 (20.0%)	3 (25.0%)
*HLA-C*07:01*	7 (35.0%)	0 (0.0%)
*HLA-C*07:02*	5 (25.0%)	0 (0.0%)
*HLA-C*08:02*	1 (5.0%)	1 (8.3%)
*HLA-C*16:01*	0 (0.0%)	1 (8.3%)

Supplementary Table S3 The table summarizes HLA-A, -B and -C allele frequencies in PsA patients and healthy controls. The number of individuals carrying the allele is not listed. *Abbreviations: HLA, human leukocyte antigen; PsA, psoriatic arthritis*.

ID	HLA-Alleles					
PsA-1	A*02:01	A*24:02	B*07:02	B*18:01	C*07:01	C*07:02
PsA-2	A*02:01	A*03:01	B*07:02	B*35:01	C*07:01	C*07:02
PsA-3	A*01:01	A*31:01	B*07:02	B*15:01	C*06:02	C*07:02
PsA-4	A*01:01	A*01:01	B*08:01	B*08:01	C*07:01	C*07:01
PsA-5	A*01:01	A*02:01	B*08:01	B*08:01	C*07:01	C*07:01
PsA-6	A*02:01	A*68:01	B*08:01	B*50:01	C*06:02	C*07:01
PsA-7	A*02:01	A*02:05	B*27:02	B*50:01	C*02:02	C*06:02
PsA-8	A*03:01	A*68:02	B*07:02	B*14:02	C*07:02	C*08:02
PsA-9	A*02:01	A*02:01	B*15:01	B*27*02	C*02:02	C*03:03
PsA-10	A*01:01	A*03:01	B*07:02	B*13:02	C*06:02	C*07:02
HC-1	A*01:01	A*02:01	B*15:01	B*37:01	C*03:03	C*06:02
HC-2	A*01:01	A*24:02	B*15:01	B*27:05	C*02:02	C*03:03
HC-3	A*02:01	A*03:01	B*51:01	B*51:01	C*01:02	C*05:01
HC-4	A*01:01	A*24:02	B*15:01	B*57:01	C*03:03	C*06:02
HC-5	A*02:01	A*02:01	B*44:03	B*44:05	C*02:02	C*16:01
HC-6	A*02:01	A*32:01	B*13:02	B*14:02	C*06:02	C*08:02

Supplementary Table S4 HLA-A, -B and -C genotypes of all PsA patients and healthy controls included in the study.

Antibody	Fluorophore	Clone	Supplier	Cat. No.
CD3	Pacific Blue	UCHT1	BioLegend	#300431
CD4	Brilliant Violet 510	SK3	BioLegend	344634
CD8	PE/Cy7	SK1	BioLegend	#344712
CD14	Alexa Fluor 488	HCD14	BioLegend	#325610
CD16	PerCP/Cyanine5.5	3G8	BioLegend	#302028
CD19	APC/Cy7	HIB19	BioLegend	#302218
HLA-A, B, C<br>(MHC I)	PE	W6/32	BioLegend	#311406

Supplementary Table S5 Markers for measuring MHC class I cell surface expression on CD4+ and CD8+ T cells, B cells, monocytes and neutrophils by multicolor flow cytometry. Fluorophore, Clone, Supplier and Cat. No. are listed.

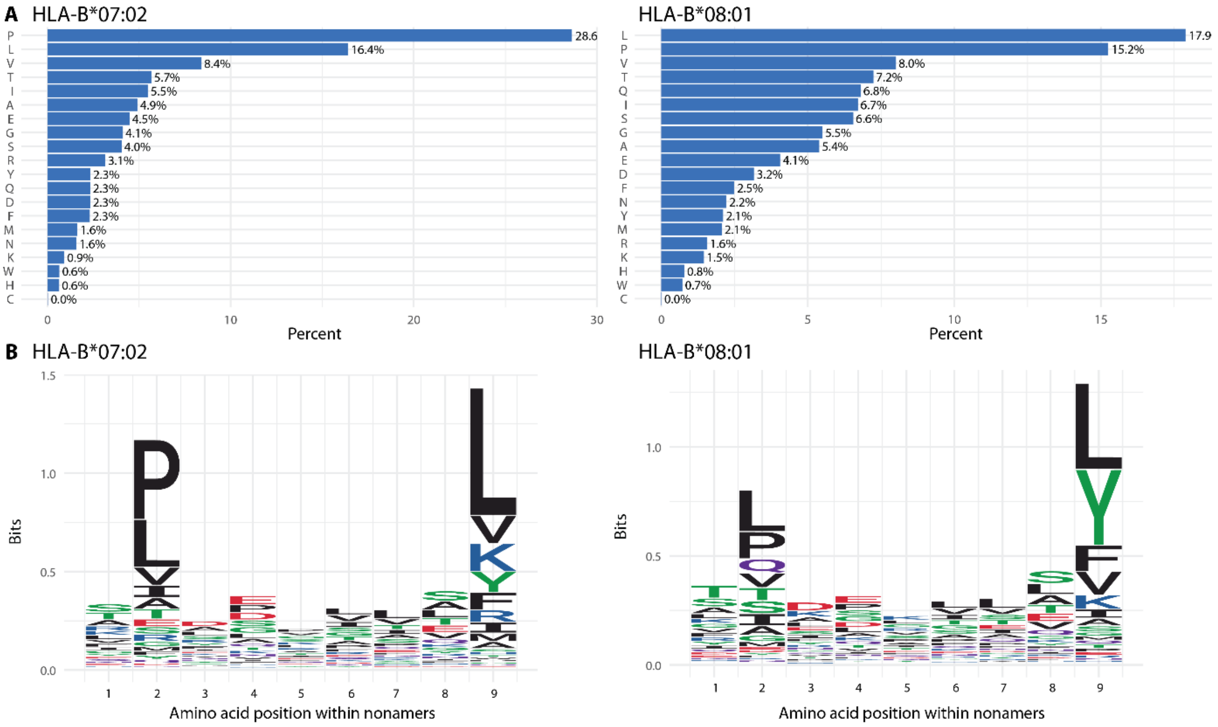

Supplementary Figure S3 Control immunopeptidome analysis confirms that neither *HLA-B*07:02* (n=4 healthy controls) nor *HLA-B*08:01* (n=4 healthy controls) exhibits increased glutamic acid at position 2. (A) Frequency of amino acids (one letter amino acid code) at position 2 of MHC class I-bound peptides. (B) Sequence logos of all identified nonamers. The size of the letter represents the relative abundance of each amino acid. The sequence logos use a chemistry-based color code in which acidic residues are shown in red, basic residues in blue, polar uncharged residues in purple, hydrophobic residues in black, and aromatic residues in green. *Abbreviations: MHC, major histocompatibility complex.*

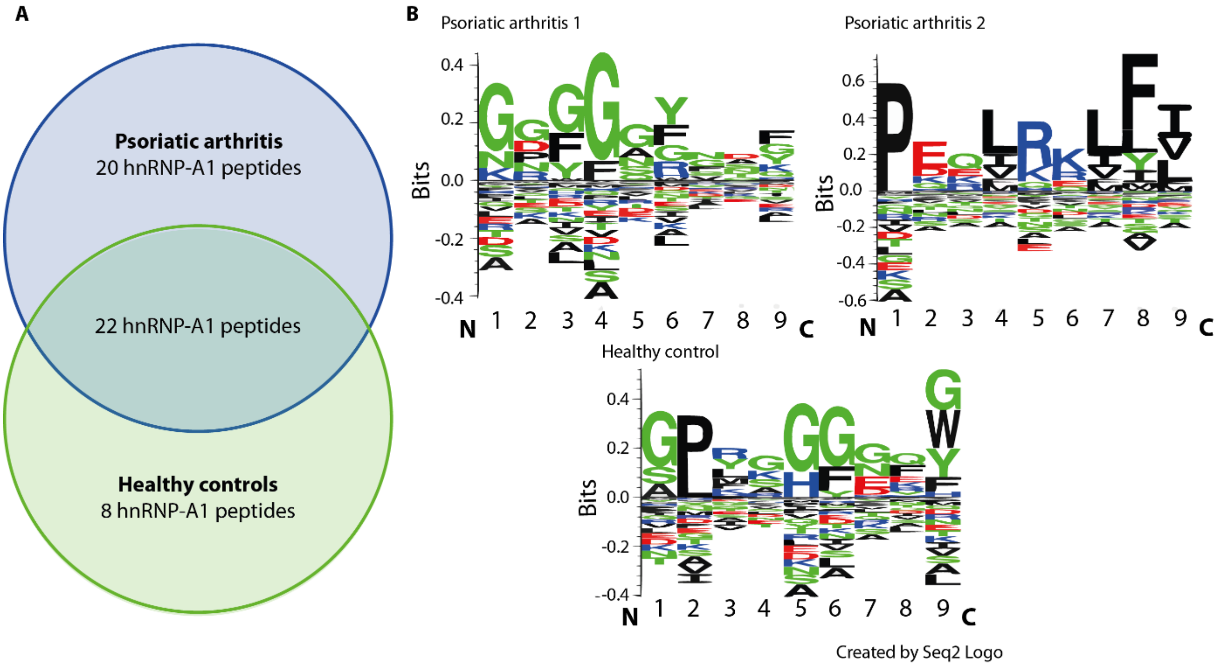

Supplementary Figure S4 Number of (hnRNP A1) peptides in MHC class I immunopeptdomes and sequence logos of hnRNP A1 nonamer clusters. (A) Venn diagram showing the number of hnRNP A1 peptides shared and those unique to PsA patients and healthy controls, respectively. (B) Sequence logos (one letter amino acid code25) of hnRNP A1 nonamer clusters in PsA patients (top) and healthy controls (bottom). The relative abundance of each amino acid is represented by the size of its letter. In PsA, higher relative abundance of glutamic acid at position 2 was identified in cluster 2 as was the PEQLRKLFI motif, which was not present in healthy controls. Sequence motifs of hnRNP A1 peptides found in the MHC class I heterogeneous nuclear ribonucleoprotein A1 immunopeptidome were created using GibbsCluster.
